# The wellbeing framework for consumer experiences in the circular economy of the textile industry

**DOI:** 10.1057/s41599-025-05813-9

**Published:** 2025-09-29

**Authors:** Bruna Petreca, Carey Jewitt, Aikaterini Fotopoulou, Lili Golmohammadi, Ricardo O’Nascimento, Lucy Chamberlin, Nadia Bianchi-Berthouze, Marianna Obrist, Sharon Baurley

**Affiliations:** 1 Royal College of Art, London, UK; 2 University College London, London, UK; 3 King’s College London, London, UK; 4 Norwegian University of Science and Technology, Trondheim, Norway

**Keywords:** Psychology, Sociology

## Abstract

Conspicuous consumption, driven by immediate satisfaction, novelty, and status, contradicts the Circular Economy’s (CE) goals of reducing consumption and waste. As the CE evolves into a global mission supported by legislation, it must address overconsumption by adopting a humanist, design-focused, participatory approach that fosters alternative cultures of consumption. This paper investigates the potential of leveraging human wellbeing as a strategic approach to achieving circular sustainable consumption of textiles. It proposes that strengthening the connection between human wellbeing and material resource flow, particularly through a garment’s lifecycle, can aid in reducing the textile consumption necessary for a successful CE. The ‘Wellbeing framework for consumer experiences in the circular economy of the textile industry’ positions consumer wellbeing as essential for the circular value chain of textiles. It serves as a cornerstone for designing consumer experiences that support a CE, informs alternative narratives for the industry and society, and has the potential to influence policy. The Framework is grounded in a comprehensive literature review examining how consumer wellbeing can drive the social health benefits of circularity, foster new sustainable consumption cultures, and serve as a consumer-centric tool for achieving zero waste through responsible and personalised engagement with consumption, reuse, and recycling. The iterative literature review and interdisciplinary elaboration followed five stages: review, selection, empirical testing, synthesis, and abstraction to achieve the final framework. The Framework comprises 16 wellbeing dimensions clustered into three categories: being well, feeling well and doing well. The primary contribution of this framework is its holistic approach to integrating and balancing the hedonic and eudaimonic dimensions of wellbeing within the context of the CE. It conceptualises wellbeing as a dynamic temporal process that evolves throughout the consumption journey, encompassing moments of both satisfaction and challenge, and addresses social factors such as the embodied experiences and self-perception elicited by a garment.

## Introduction

This paper argues that achieving a stronger coupling of human wellbeing and material resource flow (i.e. the lifecycle phases of a garment) can contribute to realising the global reduction of textile consumption for a successful Circular Economy (CE). Whilst acknowledging that sustainability is a complex systemic economic, social, and political issue that extends beyond the power of individual consumers to resolve, consumer behaviour, awareness and attitudes have an important role in future CEs of textiles. Specifically, we argue that consumer behaviour is a vital component within the framework for the circulation of products and materials (Wastling et al., 2018). This paper reviews existing literature to consider the potential to amplify wellbeing through the design of alternative circular consumer experiences and how this might be developed as a strategy to slow down fashion consumption (i.e. fashion consumption is deeply embedded in our society, merging personal expression, economic progress, and environmental effects). The constant demand for and access to new clothing, characteristic of ‘fast-fashion,’ has created a major issue—overconsumption. In the UK and the EU, the average person consumes over 25 kg of textiles annually, 2 to 3 times the global average (Niinimäki et al., 2020), with clothing amounting to more than 60% of this (EEA, 2019; WRAP, 2019).

The Textiles Circularity Centre seeks to turn post-consumer textiles, crop residues, and household waste into renewable materials for use in fashion apparel by developing new supply chains, textile production methods, and consumer experiences. This paper seeks to better-define wellbeing in the context of circular textile consumption to support the design of related future consumer experiences, products, and services. Wellbeing is a complex term; while frequently used, it is seldom defined, often vague, and empirically lacking. One consequence of this is that wellbeing is mobilised for many different purposes across competing and contradictory sectors of fashion. It is a common feature of fast-fashion discourses, with many large brands integrating the vocabulary of sustainability, recycling, the CE, and wellbeing in their marketing direct and indirect greenwashing (Badhwar et al., 2024). For example, the manager of HandM global sustainability stated “We have had the power to create desire; I think we also have the power to create meaning and well-being” (Magnus and Toriello, 2020). This reflects wider trends in the evaluation of wellbeing which show that the term ‘subjective wellbeing’, broadly referring to how individuals understand and evaluate their lives, has been rising steadily for over two decades (Barrington-Leigh, 2022).

Conceptualisations of subjective wellbeing often distinguish between ‘hedonic’ wellbeing (short term pleasure-seeking associated with fashion consumption), and ‘eudaimonic’ wellbeing (longer-term, agentive ways of living). In this paper, we propose a holistic understanding of wellbeing within the context of the textile CE that repositions hedonic wellbeing and incorporates eudaimonic facets. This aligns with research suggesting that a combination of hedonic and eudaimonic aspects is essential for a good life (Huta, 2016). To realise a holistic understanding, we assembled an interdisciplinary, human-centred team of experts in social sciences, psychology, neuroscience, human-computer interaction, and design. Taking a phenomenological approach that views individuals, society, and the environment as integrated, we contribute to the current discourse by providing a more situated and relational perspective than other human-centred or positivist approaches.

This paper makes two key contributions. First, it consolidates the literature on the intersection of wellbeing, sustainability, and the CE. Second, it unpacks, defines and explores the concept of wellbeing through a set of dimensions identified as crucial for sustainable consumption. We conclude that the set of wellbeing dimensions presented here are a useful step towards their operationalisation, as these contribute to understanding how wellbeing might be better coupled with the circular resource flow and help to narrow the gap between consumer behaviour and the CE.

## Background

### Consumption (and the need to reduce it)

Research has highlighted the lack of focus on consumers and models of consumption in a CE (Camacho-Otero et al., 2018; Camacho-Otero et al., 2020; Kirchherr et al., 2017). Concepts such as sufficiency, or ‘enoughness’ (Princen, 2005) are integral to the CE yet remain inadequately represented in its discourse (Bocken et al., 2022; Bocken and Short, 2020). Consumers (their behaviours and perceptions) critically influence material and product longevity, reuse or disposal (Cooper, 2005; Fletcher, 2012; Mugge, 2017). They have been described as the ‘most central enabler of CE business models’ (Kirchherr et al., 2017, p. 228), and are at the centre of the Ellen MacArthur Foundation’s seminal ‘butterfly diagram’. However, only 19% of CE definitions include consumption (Kirchherr et al., 2017), and there has been a notable lack of theoretical or empirical research on this topic to date (Camacho-Otero et al., 2018; Camacho-Otero et al., 2020; Kirchherr et al., 2017).

From Veblen’s 1899 critique of conspicuous consumption (Veblen, 1994) to Packard’s mid-20th century commentaries on waste and manipulative advertising, and the social and cultural theories of Baudrillard, Giddens, and Bourdieu in the 1980s and 1990s, the subject of consumption and the figure of the consumer have historically been controversial and contested (Gabriel and Lang, 2006). Consumers have been viewed both as rational agents, and victims of corporate manipulation; characterised as creative bargain hunters, hedonistic pleasure seekers, fantasists, social or symbolic communicators, and identity builders (Gabriel and Lang, 2006; Julier, 2013; Kjellberg, 2008), who express themselves through products that become extensions of themselves (Belk, 1988).

Recently, the division between production and consumption has also become blurred by the introduction of the ‘prosumer’, where customers actively participate in creating brands and artefacts (Ritzer, 2014; Ritzer et al., 2012). Economically, consumption has become a measure of growth and success, with shopping deemed a virtuous activity essential for economic development (Baudrillard, 1998; Gabriel and Lang, 2006; Shiller, 2012), fulfilling human needs for creativity, meaning and identity (Jackson, 2005a). However, consumption also has negative connotations, implying wastefulness or destruction, especially regarding planetary resources (Røpke, 2009), as well as the commodification of relationships and social values, leading to feelings of alienation, meaninglessness and negative health consequences (e.g. obesity). Despite the wellbeing advantages of wealth, higher incomes have a complex relationship to wellbeing and social inequality across the globe (Richins, 2013; Sandel, 2012; Ward et al., 2016; Killingsworth et al., 2023).

### Wellbeing, sustainable consumption, and CE

Sustainability is a complex systemic issue, understood as the need to keep a ‘safe operating space for humanity’ (Rockström et al., 2009) within planetary boundaries. The CE is a recent sustainability paradigm (Geissdoerfer et al., 2017), and offers one practical way forward with its emphasis on ‘closing the loop’. The Textiles Circularity Centre promotes a CE model that redefines material value beyond cost to encompass socioeconomic factors. This model focuses on significantly reducing consumption; extending garment use and lifespan; promoting reuse and recycling; using traceable and durable materials; localising production; minimising or eliminating the use of virgin materials; and reducing land use. Consumers are central to this model, yet their role in resource usage and allocation has often been overlooked in CE conceptualisations, which prioritise resource flows. The Textiles Circularity Centre addresses this gap by investigating ways to support consumers in adopting more ‘circular’ practices.

While sustainability is beyond the power of individual consumers to resolve, sustainable consumption involves utilising services and products that satisfy needs and enhance quality of life, while minimising environmental impact to ensure the wellbeing of future generations is not compromised (Vargas-Merino et al., 2023). As the textiles and fashion industry increasingly focuses on sustainability, a significant gap still exists in integrating human wellbeing into standard frameworks that purport to measure sustainability, such as Life Cycle Assessment (LCA). LCA is a ‘bottom-up’ method that analyses processes of extraction, production, use and disposal associated with a production system, aggregating contributions from each sub-process and allocating environmental impacts per “functional unit” (e.g. carbon emitted per kg of fibre, or water used per shirt). It is widely used to assess the environmental impacts of textiles, particularly in the production stages (e.g. Amicarelli et al., 2022; Yan and Song, 2021). It largely overlooks the multidimensional nature of wellbeing, including physical, psychological, and social dimensions, which are critical in evaluating the impacts of fashion consumption. Recent developments in Social Life Cycle Assessment (S-LCA) have begun incorporating indicators related to worker health, autonomy, and social equity (Zamagni et al., 2011; Ekener and Moberg, 2023). Fashion-specific studies show how S-LCA can reveal hidden social risks across global textile supply chains (Muñoz et al., 2023) and assess contributions to broader sustainability goals, including wellbeing (Jørgensen et al., 2020). However, these approaches remain limited in scope and uptake, and of particular relevance to this paper is that the environmental impacts of consumption and the human scope remain under-researched.

Despite the well-established negative impact of fashion on the planet and garment workers (EAC, 2019), many consumers remain largely unaware of these issues. The prevailing linear model does not foster a prosumer attitude (Petreca et al, 2022). Even when consumer awareness improves, it often has little effect on consumption patterns and behaviours (Goworek et al., 2012; Becker-Leifhold, 2018; Niinimäki, 2010; Nielsen et al., 2022). Existing approaches to changing consumer behaviour are primarily normative; relying on educational and behavioural messaging, interventions such as nudges, and efforts to shift beliefs, decisions, and ‘habits’ away from ‘bad’ consumption and towards pro-environmental ‘good habits’ that support long-term sustainability goals. Unsurprisingly, changing labels, providing information, using scare tactics, or trying to instil a sense of ‘sacrifice’ have shown limited impact (Wilk, 2022; Bly, Gwozdz and Reisch, 2015; Jackson, 2009). Additionally, the ‘double dividend’ concept, which suggests that reducing material consumption benefits both the environment and individuals (Jackson, 2005b), does not hold true (Chamberlin and Callmer, 2021). Despite the negative association between materialism and happiness (Layard and De Neve, 2023; Dittmar et al., 2014; Richins, 2013; Jackson, 2005b), material goods are integral to how individuals negotiate values, shape identities, and express social and personal meaning, making behavioural change particularly challenging (Jackson, 2005b). Moreover, the relationship between income, consumption and wellbeing varies depending on the inequality of the society under consideration and position of individuals in this spectrum (Buttrick and Oishi, 2023; Killingsworth et al., 2023).

Challenging the problematics of current metrics applied to the study of human development, particularly in how they prioritise economic growth over wellbeing (Max-Neef, 1995), Manfred Max-Neef proposed a taxonomy of Fundamental Human Needs to override economic indicators like GDP (Max-Neef, 2017). The Fundamental Human Needs include categories such as subsistence, protection, affection, understanding, participation, leisure, creation, identity, and freedom. While Max-Neef’s framework has been influential in various policy discussions, our research shows that there has been limited direct application of his taxonomy to the specific issue of fashion overconsumption. The fashion industry’s overconsumption problem is often discussed in terms of environmental and social impacts, but integrating Max-Neef’s human needs perspective could provide a more comprehensive understanding when rethinking clothing consumption (Eräpuu, 2024).

Connecting wellbeing to sustainable consumption involves addressing several challenges. Engaging consumers through hedonistic or extrinsic values can backfire, reinforcing self-enhancement behaviours (PIRC, 2011). Evans’s framework (2019) of six ‘moments’ of consumption^1^ highlights that disposal and waste are inevitable counterparts to consumption. Moreover, consumption often results from daily practices such as washing, eating, and dressing, which are significantly influenced by commercial interests (Kuijer et al., 2013; Evans, 2019). The distinction between conspicuous and inconspicuous consumption adds complexity (Evans, 2019; Welch et al., 2017). Additionally, ‘competent’ consumers must engage in disposal processes, including divesting from accumulation (Gregson et al., 2007). This multifaceted approach underscores the complexity of fostering sustainable consumption habits that align with wellbeing.

A growing body of research suggests that addressing more situated aspects of human wellbeing – such as enjoyment and pleasure, self-expression, and community – may effectively promote sustainable consumption (Chamberlin and Callmer, 2021). As Wilk notes, people ‘need assurance that a more sustainable world is not going to be bland, boring and full of sacrifice’ (Wilk, 2022, p. 265). To mobilise this strategy, wellbeing and the circular experiences and services that support it, need better definition in the context of textile circularity. Our focus on wellbeing addresses the sustainability literature’s idea that emotional engagement, enjoyment, and self-expression are more effective in reaching people than information (e.g. labels and measurements), sacrifice, moralistic criticism, or suppressing personal or social needs (Chamberlin and Callmer, 2021). Notably, wellbeing, albeit in a reduced and partial form, is already liberally mobilised by fashion brands and retailers to maximise consumer engagement and demand. McKinsey estimates the global wellness market size to be above $1.5 trillion, with annual growth of 5 to 10 percent (Callaghan et al., 2021). To this end, we ask, *how might a holistic notion of human wellbeing be mobilised differently for sustainable circular consumption within a capitalist system?*

The link between hedonic wellbeing (pleasure-seeking), conspicuous consumption, and fashion is well-established. In capitalist economies, a hedonic perspective on wellbeing has dominated, viewing wellbeing through ‘the lens of individual purchasing power rather than overall social outcomes’ (Brand-Correa and Steinberger, 2017, p. 44). Contemporary hedonism has serious consequences for sustainability, as any limits on consumption (e.g. resource use) can be perceived as limits to wellbeing from a mainstream economic perspective (ibid). Research on fast-fashion shows that hedonic tendencies and ‘brand love’ significantly affect impulsive buying (Sari and Yasa, 2021), and increase the positive affect that consumers feel when visiting stores, further stimulating impulse-buying (Liapati et al., 2015).

There have been efforts to reposition hedonic wellbeing for sustainability, including Hedonistic Sustainability (proposed by architect Bjarke Ingels, Mohtadi, 2016), and Eco-hedonism or Alternative Hedonism (Soper, 2020; 2008). Chamberlin and Callmer (2021) argue that ‘Alternative Hedonism’ highlights the pleasures of rethinking and changing consumption patterns, suggesting that the “good life” can be both seductive and virtuous. While highlighting the negative aspects of consumerist culture can diminish its appeal, it is crucial that people perceive alternatives as viable, available, and attractive (Vargas-Merino et al., 2023). Aesthetics is reportedly crucial for consumer choices, even when material sustainability is emphasised in a retail experience (Petreca et al., 2022). ‘Green perceptions’ related to effort, trust, and preconceptions are critical barriers maintaining the gap between consumers’ intentions and purchasing behaviour (Johnstone and Tan, 2015). This reconceptualisation of hedonism arises from the need to challenge the notion of sustainability as a hardship, sacrifice, or compromise that must be ‘endured’ for the greater good—a notion that few people will embrace and that does not work.

In contrast to the short-termism of hedonism, eudaimonic wellbeing (less often associated with fashion consumption) represents the long-term achievement of active, agentive ways of living with embodied consequences and social meaning that cannot be reduced to momentary affective experiences. Eudaimonia encompasses the broader notion of a life full of purpose and potential, underpinned by the power to make choices and participate in one’s own life.

While hedonic wellbeing emphasises the individual, eudaimonia acknowledges the importance of everyday social practices, social norms, and intercultural and intergenerational factors in wellbeing. This situates people’s wellbeing within the broader societal context, highlighting the role of social limits on individual choice (Brand-Correa and Steinberger, 2017). It also draws attention to the role of social institutions and systems in enabling individuals to flourish. Summed up by ‘the good life’ (Doyal and Gough, 1991), eudaimonic wellbeing goes beyond subjective experiences to focus on ‘what one can do or be in one’s life’ (O’Neill, 2008, p. 165). It includes aspects such as self-discovery, perceived development of one’s best potentials, a sense of purpose and meaning in life, intense involvement in activities, investment of significant effort (‘effortful engagement’), and enjoyment of activities as personally expressive (Waterman, 2024).

## Method

In this paper, we propose a holistic approach to wellbeing and the CE that integrates and balances the hedonic and the eudaimonic dimensions of wellbeing. This approach aims to move arguments, interventions, and policy beyond notions of wellbeing as short-term pleasure, in which effort and ethics are always a negative cost. It also shifts the debate from a sole focus on individual behaviour, choice, and responsibility to better understand the social factors and norms that shape people’s everyday practices, including patterns of consumption.

From a holistic perspective, wellbeing is understood as a dynamic temporal process that unfolds during consumption (Evans, 2019). This process encompasses moments of both satisfaction and challenge, and is deeply connected to social factors such as the embodied experiences and self-perception evoked by a garment. To achieve this comprehensive understanding, we convened a highly interdisciplinary, human-centred team, comprising experts in social sciences, psychology, neuroscience, human-computer interaction, and design. Our approach adopts a phenomenological stance, viewing individuals, society, and the environment as integrated entities. We examine issues from the micro, individual level to the macro, socio-environmental level.

While the terms wellbeing and its relationship to sustainable consumption are somewhat nebulous and poorly defined, it is frequently mobilised to encourage fashion consumption. Thus, we set out to define wellbeing to support sustainable consumption practices. Working iteratively with literature review and interdisciplinary elaboration, the stages outlined below were followed:

**Literature review:** A comprehensive literature review on wellbeing, consumption, CE, and sustainability was conducted to identify candidate wellbeing concepts. The literature was reviewed across various disciplines, reflecting the research team’s specialisms, including sustainability, design, fashion, healthcare, sociology, psychology, and neuroscience. The review process involved formal searches across the databases Scopus, Web of Science, and Google Scholar, as well as key fashion and sustainability journals, and industry and policy documents. We used combinations of the search terms *wellbeing, textiles, fashion, co-creation, participation, embodiment, sensory, sustainability, consumption*, and *circularity*. This was followed by a second more specific search using the wellbeing terms and concepts associated with fashion and circularity that emerged from the initial search (e.g. attachment, care). Additional literature was identified by following references from initial sources and through discussions among the team (a snowballing process), which uncovered further relevant texts.

**Interdisciplinary selection:** The literature was shared and discussed across the team to filter, select and synthesise the concepts relevant to wellbeing in the context of fashion-textiles, sustainability and circularity. This stage generated 24 wellbeing concepts.

**Filtering key concepts empirically:** We carried out empirical testing to filter the key concepts that are most meaningful to consumers (Jewitt et al., in review). We narrowed down to 16 interconnected dimensions of wellbeing. This work was part of the creation of the Circular Experience Toolkit, which is designed to prompt and foster interdisciplinary, design-led conversations with and between researchers, textile and fashion industry professionals, and consumers to support the design of meaningful circular consumer experience. We report on the empirical testing and development of the Toolkit elsewhere (Jewitt et al., forthcoming). Here we include this stage solely as part of the methodology for developing the wellbeing framework. The toolkit contains Wellbeing cards, which consist of the concepts selected from the literature. We tested these 24 concepts-cards through studies at the Regenerative Fashion Hub (October–November 2022, *n* = 45) to explore how, when, and to what extent the wellbeing dimensions featured in participants’ experiences. Insights from the analysis were used to refine the cards, adding or editing out some of the Wellbeing dimensions, and amalgamating or renaming others. For example, we added ‘Effortful’—a prominent feature of the data and combined ‘Agentive’ with ‘Sense of Control’. Through this process, we selected and consolidated 16 Wellbeing dimensions (cards). Table 1 shows the results of this iterative work.

**Table 1:** Results of the iterative work on the wellbeing dimensions.

*Wellbeing* dimensions from literature^a^	Consolidated wellbeing dimensions^b^
Affordable	Affordability
Agentive	Agentive and sense of control
Sense of control	----- *(folded into the above)*
Attuned and in touch	----
Sense of attachment	Attachment
Sensuous	Bodily and sensory
Caring	Caring
Sense of connection	----
Sense of belonging	Community and belonging
Competence (sense of ‘mastery’)	Competence (sense of ‘mastery’)
Confidence	
Creative	Creativity and self-expression
Expressive	----- *(folded into the above)*
	Effortful
Engaged	Engagement (participation ………… collaboration)
Participation	----- *(folded into the above)*
Enjoyment and pleasure	Enjoyment and pleasure
Feel great	
Self-discovery	Learning
Motivation	-----
Optimism	Optimism
Ownership	-----
Purposeful	-----
	Playfulness
	Future thinking (Prospective self)
Self-esteem	Self-worth
Sense of satisfaction	

^a^Left column shows the 24 dimensions we tested empirically.

^b^The right column shows the 16 consolidated dimensions following our analysis of data from studies.

**Synthesising definitions:** The team worked collaboratively in synthesising definitions for the selected wellbeing dimensions situated in the context of fashion-textiles sustainable circular consumption.

**Abstracting wellbeing categories:** The final stage involved organising the 16 wellbeing dimensions under three well-established overarching categories as a framework to mobilise the potential of wellbeing to support sustainable consumer experiences. The intersection between wellbeing, sustainability, and fashion is a complex space; while we have clustered these wellbeing dimensions into three categories, they feed into and interconnect with each other in multiple directions. Indeed, these concepts are not suggested as unique and mutually exclusive categories of wellbeing, but rather as key interconnected dimensions that could guide human needs in future CE efforts.

### The wellbeing framework for consumer experiences in the circular economy of the textile industry

Research on wellbeing and sustainable fashion consumption is still in the conceptual stage, with most studies being small scale. Nonetheless, there is sufficient evidence to support the argument that consumer experiences designed around human wellbeing can significantly contribute to more sustainable consumption. We introduce the ‘wellbeing framework for consumer experiences in the circular economy of the textile industry’, which positions consumer wellbeing as a pivotal element in the circular value chain. The framework is grounded on a comprehensive literature review that examines how consumer wellbeing can drive the social health benefits of circularity, foster new consumption cultures that encourage sustainable behaviour, and serve as a consumer-centric tool for advancing towards zero waste through responsible and personalised engagement with consumption, reuse, and recycling. The framework comprises 16 factors, clustered into three overarching categories: *being well*, *feeling well* and *doing well*, which are presented in Table 2 and described in detail in the following sections.

**Table 2:** The wellbeing framework for consumer experiences in the circular economy of the textile industry.

Overarching dimensions of human wellbeing	Sub-dimensions of human wellbeing
FEELING WELL: Subjective Dimensions of Wellbeing -*Including Hedonic Dimensions and Psychosocial Needs Satisfaction*	ENJOYMENT and PLEASURE *Satisfaction, including* BODILY and SENSORY *Comfort*
	*Avoidance of negative emotions, including* BODILY and SENSORY *Discomfort*
	ATTACHMENT COMMUNITY and BELONGING CARING *Relatedness*
	SELF-WORTH *Esteem / Respect / Recognition and Power*
DOING WELL: Subjective Dimensions of Wellbeing -*Including Eudaimonic Dimensions and Personal Needs Satisfaction*	AGENTIVE and SENSE OF CONTROL EFFORTFUL ENGAGEMENT (PARTICIPATION, COLLABORATION) *Ownership / Autonomy and Active Engagement*
	COMPETENCE (SENSE OF MASTERY) LEARNING *Epistemic Mastery*
	CREATIVITY and SELF-EXPRESSION *Meaningfulness / Purpose / Authenticity* PLAYFULNESS
	OPTIMISM FUTURE THINKING (PROSPECTIVE SELF) *Self-efficacy*
BEING WELL: Objective and Socioeconomic Dimensions of Wellbeing	AFFORDABILITY *Practicality/Feasibility/Ease of Access or Use* *Material and Basic Needs Satisfaction (a key aspect of human wellbeing, though not a focus of CX)*
	*Health-promoting conditions, experiences, and behaviours (see feeling well and doing well)*

### Feeling well

Feeling well refers to the subjective dimensions of wellbeing, including hedonic dimensions and psychosocial needs and satisfaction. We include six wellbeing concepts within this category: enjoyment and pleasure, bodily and sensory experiences, attachment, community and belonging, caring, and self-worth, each of which is defined and situated in the context of textile circularity below.

***Enjoyment and pleasure***, defined as the state or experience of positive emotions and sensory pleasure, are significant features of wellbeing. Pleasure is related to and generated through the process of consumption (Evans, 2019), such as the pleasure of trying on clothes with friends, dressing up to go out, or making a garment. Pleasure also relates to the multisensory and physical experiences of a garment that fits well, feels great, and looks good, and to experiences of comfort and other physical-emotional aspects, including increased self-esteem, social belonging and attachment. A garments’ functionality, emotional, and ‘aesthetical experiences’ (design, style, colour, and material choices) contribute to the pleasure of garments (Niinimäki and Armstrong, 2013, p. 193). As such, enjoyment and pleasure are an important means by which people bond emotionally with garments, forming a ‘conduit for attachment’ (Niinimäki and Armstrong, 2013, p. 192). Enjoyment and pleasure are also key aspects of experimenting with renting clothes, such as trying different styles and diversifying one’s wardrobe, and are major reasons why consumers opt into this collaborative consumption practice (Becker-Leifhold, 2018). People who engage in sustainable fashion consumption have also found it ‘a source of pleasure and wellbeing,’ which they relate to a newfound sense of self-confidence, subtle resistance, and freedom from pressures to keep consuming (Bly et al., 2015, p. 127).

The wellbeing effects of ***bodily and sensory*** awareness are well established (e.g. in relation to mindfulness and somatic therapies). Clothing comfort research has extensively explored sensory and bodily awareness as a major research area in fashion-textiles and e-textiles. Bodily and sensory factors are key facets of the textiles that make up our clothes; these can significantly impact the embodied experiences of sustainable fashion consumers (Ciaunica et al., 2021; Entwistle, 2023; Sampson, 2018; Kuusk et al., 2018). The concept of ‘comfort’ in fashion studies is often seen as a physiological and psychological state of being (Höppe, 2002; Zhang and Zhao, 2008), influenced by a range of multisensory factors, including the feel, fit, texture, pressure and look of clothing that one may be wearing (Kamalha et al., 2013). For example, body temperature, clothing texture, and the tactile sensations of wetness all contribute to overall comfort levels (Havenith et al., 2002). Moreover, while social theorists have traditionally emphasised the constitution of the enclothed body through cultural systems of fashion signification (i.e. the enclothed body as image or text), there is a growing interest in the corporeal dimensions of fashion (i.e. the enclothed body as sensing, feeling and acting, e.g. Young, 1994; Entwistle, 2000; Sweetman, 2001; Negrin, 2012; Adam and Galinsky, 2012; Fleetwood-Smith et al., 2019). Interestingly, some sustainable fashion consumers now cite the bodily benefits of natural materials as a key purchasing motivation, often perceived as more comfortable to wear and better for health and skin wellbeing due to fewer chemicals or pesticides used in their production (Lundblad and Davies, 2016, p. 156). There are also benefits to engaging people reflexively with their sensory and material environments as a route to reduced consumption. For instance, the process of decluttering (e.g. using the KonMari method) has been shown to enhance confidence in what individuals value and find joy in, prompting a desire to consume less and more reflexively in the future (Chamberlin and Callmer, 2021). This signals a potential route through the bodily and sensory to the CE (ibid.). In repair, the relevance of sensory training and attuning has been acknowledged as critical both for individual practices (e.g. for understanding materials), as well as for communal spaces where sharing sensibilities is critical for developing a repair practice (e.g. learning skills and sharing taste with others) (Durrani, 2021).

Fostering consumers’ ***sense of attachment to garments*** is another argument for reducing consumption. The idea is that emotional attachment to a product encourages care, thereby extending its lifespan and reducing the likelihood of purchasing replacements (Maldini and Balkenende, 2017; Niinimäki and Koskinen, 2011; Norman, 2005). These concepts are underpinned by the longer-standing theory of ‘emotionally durable design’ and stages of attachment formation (Chapman, 2005) from industrial design. Researchers propose design frameworks and strategies to nurture stronger attachment relationships between people and products to curb overconsumption (Mugge et al., 2005; Chapman, 2005; Norman, 2005). This idea connects to the notion that *effort* (discussed later) may play a crucial role in generating the link between attachment and care, a topic explored to a lesser extent in both this and fashion consumption literature. We use the term “attachment” to refer to a sense of connection and affection towards something. Designing for meaningful ‘attachment elements’ and ‘product satisfaction dimensions’ (Niinimäki and Koskinen, 2011) is challenging because individuals have distinct histories, expectations, tastes, and experiences. To date, co-design - where consumers are involved in the design, personalisation, and/or manufacture of products—has emerged as a prominent and promising strategy for fostering attachment to garments (Maldini and Balkenende, 2017) and other types of products (Norton et al., 2012; Franke et al., 2010; Norman, 2005). While some studies have explored this link in the context of fashion retail, suggesting that online configurators can generate attachment (Kim and Lee, 2020; Teichmann et al., 2016), long-term empirical evidence is still needed (Maldini and Balkenende, 2017). Another route to attachment established in the literature is through repair. Emotional attachment to products can significantly influence people’s decisions to repair and care for items (Lee and Wakefield-Rann, 2021; Terzioglu, 2021; Mugge et al., 2005). Attachment can also come *from* the repair experience itself (Korsunova et al., 2023), where the effort and time invested add value to products. For instance, the effort involved in mending or making has been shown to provide a sense of reward and personal achievement (Niinimäki and Armstrong, 2013). Furthermore, the practice of repair can be experienced as ‘enjoyable and empowering’ (Korsunova et al., 2023, p. 7). Thus, creating positive repair experiences appears to be a valuable route to fostering attachment both to the repaired garment and to the practice of repair itself (Durrani, 2021).

The politics and economics of ***caring***, associated with attachment and wellbeing, have been further explored through ideas around slow movements, emotionally durable design, and meaningful design (Chapman 2021; 2015; Casais et al., 2016; Sheth et al., 2011; Cooper, 2005), which link care for nature with care for the material world. The literature on repair most prominently reflects ideas about caring for garments, others, and the planet. However, numerous examples in the literature highlight how factors like affordability (both price and time) can override caring for garments and the planet. In ‘wardrobe examinations’ research, Heinze (2021) observed that sustainably caring for and discarding garments presents a challenge for those who are time-poor. Additionally, caring for belongings can sometimes relate to long-standing household values of thriftiness and avoiding waste, as much as to sustainability (Korsunova et al., 2023, p. 5). This is echoed by earlier research into motivations for ‘anti-consumption’ practices (e.g. rejecting, reducing, reusing, recycling) by Black and Cherrier (2010), which found that although people expressed care for the environment, ‘care for individual needs’ (such as saving money, beauty, or independence) was a much stronger aspect of anti-consumption for sustainability. This attitude is reflected in Fashion Revolution Poland’s recent survey of 4000 fashion consumers’ habits and priorities, which noted that ‘we do care, but mostly for ourselves’ (Fashion Revolution Poland, 2023, p. 6). Thus, there are gaps between the theories and practicalities of enacting caring practices.

There is a strong argument for the value of ***community and belonging*** for increasing consumer participation in the CE. Attention to how to bring people, their wellbeing, and daily practices back into the conversation around the CE, has primarily focused on the technicalities and phases of resource flows. In other words, *what does a ‘a sustainable way of living with things’* (van der Velden, 2021) *look like?* We use the term community to describe groups with shared purposes, interests, and responsibilities, ‘who respect the individual differences among members, and who commit themselves to the wellbeing of each other and the integrity and wellbeing of the group’ (Wood and Judikis, 2002, p. 12). As Taylor (2019) observes, there has been a marked increase in community groups that swap, rent, repair, and gift—supporting competence and learning (discussed later) and idea-sharing for alternative fashion economies. Sharing or swapping events, for example, can both raise awareness around issues related to overconsumption and address ‘an intense desire’ for a sense of community (Albinsson and Yasanthi Perera, 2012). Meanwhile, studies of local community repair have emphasised these as settings of material and social entanglements (van der Velden, 2021; Durrani, 2018, 2021), where people exchange stories, knowledge, ‘celebrate’ successful repairs, and gain agency over the dominant take-make-waste system through exercising their ‘right to repair’ (Hernandez et al., 2020).

Building community can contribute to feeling agentive and in control, a sense of ***self-worth***, and a sense of belonging, which relate to the notion that it is possible to challenge the dominant system of fast-fashion and the unsustainable practices that underpin it. Self-worth encompasses notions of self-esteem, respect. Rosenberg (1965) focused on the evaluative process of self-esteem, involving an attitude of approval or disapproval of the self (i.e. self-regard). Rogers emphasised the affective process of self-esteem, the “emotionalised attitudes and feelings directed toward the self” (Rogers, 1950, p. 375). For example, research by Balderjahn et al. (2020) into the effects of voluntary simplicity, collaborative consumption, and debt-free living on consumer wellbeing found these strategies did not diminish wellbeing, but instead contributed to feelings of autonomy, self-esteem, and financial stability. Whereas self-worth is typically tied to social acceptance in fashion consumption (a concern with how one is perceived by others), sustainable fashion consumers’ self-worth can be more internally driven, arising, for example, from factors such as feeling comfortable in the materials, and in being able to, and confident in, expressing non-conformist values (Lundblad and Davies, 2016). These dimensions of self-worth, and a sense of feeling agentive, particularly speak to eudaimonic wellbeing—that is, the (long-term) achievement of active, agentive ways of living with embodied consequences and social meaning which cannot be decomposed into momentary affective experiences.

### Doing well

Doing well relates to subjective dimensions of wellbeing, including eudaimonic dimensions and personal needs satisfaction. There is a strong argument within the sustainability literature that people are—or need to become—active stakeholders in the processes of fashion sustainability and circular consumption. We suggest that wellbeing can be leveraged as a route to position the consumer more actively through fostering ***agency and a sense of control***. Being an agentive consumer involves actively, independently, and competently interacting, participating, or co-creating in the process of fashion ‘consumption’. This positionality often emerges through the process of exchanging skills and resources, and challenging mainstream consumption. Such processes likewise can contribute to improved self-worth, i.e. evaluative processes regarding one’s self-concept (see self-worth, above).

***Engagement*** (ranging from participation to collaboration) is a key aspect of being an agentive consumer and wellbeing more generally. It refers to the state of being engaged with and active in a process in various forms. Participation in the CE can snowball; even if consumers’ initial motivations may not be related to sustainability, this can create a ‘virtuous circle of consumer engagement’ (for example, in the case of second-hand markets – Machado et al., 2019). Sustainable consumer practices – spanning co-design, renewal and repair, decluttering, buying less, buying second-hand, and the swapping, renting, gifting, and sharing of goods of ‘collaborative consumption’ (Botsman and Rogers, 2011) – in different ways can all serve to increase consumer engagement. For example, a study of second-hand markets found engagement to take place largely through the relationships consumers form with ‘sales’ persons and other consumers (Machado et al., 2019). Other work (ours included) focuses on developing new ways to increase consumer collaboration and participation. For example, Taylor’s ‘Ripple Effect Tool’ (2019) centres on supporting a process whereby people can participate in creating new practices, roles, functions, and values for SMEs, designers, and communities to take ‘back control of their practice and wardrobe decisions’ (Taylor, 2019, p. 415). Two associated wellbeing concepts (discussed above) that can help to achieve and support consumer participation are enjoyment and pleasure and bodily and sensory engagement.

***Effort*** is central to ‘doing well’. It has been defined as ‘the subjective intensification of mental and/or physical activity in the service of meeting some goal […] a volitional, intentional process, something that organisms apply’ (Inzlicht et al., 2018, p. 388). Effort might be considered an off-putting quality to aim for in sustainable consumption strategies, as for many, consuming ‘sustainably’ is already too effortful. However, effort is a significant part of eudaimonic wellbeing, and exerting effort purposefully has been shown to play a positive role in interactions, it is seen as “investment of significant effort in pursuit of excellence” (Waterman et al., 2010, p. 44), and is a potential bridge between wellbeing and fashion sustainability (Martela and Sheldon, 2019), in adding value in either the process or the product of effort (Inzlicht et al., 2018). For example, Lundblad and Davies’ (2016) research into the values and motivations of sustainable fashion consumers found people felt ‘good’ and a ‘sense of accomplishment’ for making more sustainable choices; a large part of this came from consumers’ effort in researching their purchases (the ‘cost of becoming informed’) before buying them. More generally, there is thorough systematic evidence showing that people subjectively value more products they either effortfully assembled, or personalised and co-designed rather than ready-made products (Eisenberger, 1992; Franke et al., 2010; Mochon et al., 2012). For instance, people are even more willing to pay for objects they laboriously create themselves, over the same objects made by experts (Mochon et al., 2012). These effects, including both the increased valuation of the effort itself, and the increased valuation of its products, are thought to arise partly due to the increased sense of control and ownership over the product which arises from the self-design process and the enjoyment people associate with their effort in these circumstances (Buechel and Janiszewski, 2014); not unlike the enjoyment and sense of accomplishment accompanying the arduous nature of completing a marathon (Hammer and Podlog, 2016).

Competence and learning are central to wellbeing and being a skilled sustainable consumer. Increasing consumer participation in the CE requires supporting consumers to become more aware and skilled, and developing the infrastructure for learning these skills. ***Competence***, or a sense of ‘mastery’, is a feature of consumers’ emotionally meaningful relationships to products (Casais et al., 2016; Grosse-Hering et al., 2013; Strauss and Fuad-Luke, 2008). That is, where they are ‘competent’ consumers, able to reflexively engage with how things are used or not used and can make informed choices about acquisition and divestment to avoid accumulation (Gregson et al., 2007). Competence can also refer to the skills and confidence required to undertake specific circular practices, such as renewal and repair, or the co-design of apparel. Interestingly, according to influential psychological theories of wellbeing, competence, is one of the three most basic psychological needs, the fulfilment of which is essential for human wellbeing (Martela and Sheldon, 2019), the other two being autonomy (the sense of volition and ownership of action) and relatedness (the sense of being in mutually caring relationships with others; Ryan and Deci, 2017).

***Learning*** is an active process of gaining skills and knowledge. Infrastructure and opportunities for learning these skills are an area for further development, but the literature highlights numerous examples that show the potential virtuous circle that can be set in motion by people learning even a relatively rudimentary skill. For example, work by Korsunova et al. (2023) shows that an introductory skill or prior experience can empower people to take on more complex (re)making or mending tasks. Durrani’s (2018) study of the learning processes and outcomes of people attending communal mending workshops found three types of learning ‘streams’ to be important: material, communal, and environmental. Participants learned skills for mending (which they were able to share with friends and family), to differentiate between ‘good- and bad-quality garments’, and expand their knowledge of garment disposal alternatives. This created a sense of community empowerment, agency, self-reliance, and caring (also featured elsewhere in our own wellbeing dimensions), and was valued as an alternative way to share knowledge (ibid).

Fashion is long-acknowledged as playing a central role in ***creativity and self-expression***, and the experimentation of identity (Händler Svendsen, 2006). Fashion allows individuals to curate their identities through stylistic choices, serving as a form of non-verbal communication. It is inherently experimental in nature as it requires and supports both designers and consumers to continually explore new forms, textures, and styles (ibid). While sustainable consumption practices are often viewed as a constraint to this process, research into sustainable fashion consumption shows that some people also use ‘anti-consumption’ as a form of creativity and self-expression: Bly et al. (2015) found sustainable fashion consumers conceptualised their consumption practices (purchasing fewer, higher-quality garments, purchasing only second hand, making/upgrading their clothes) as a ‘facilitator’ of personal style, where constraints mobilised their creativity and shifted their focus away from fashion trends. This enhancement of creativity through constraint was also observed by Taylor (2019) in her own experiment with buying nothing new for a year. Lundblad and Davies (2016) also found sustainable fashion consumers’ purchasing decisions related to expressing themselves as ‘not following the herd’ by resisting overconsumption and fast-fashion, and through ‘the unique style’ of sustainable brands, which, being small, meant their designs were less ubiquitous. In addition, Black and Cherrier’s work (2010) has highlighted how those undertaking ‘anti-consumption’ practices (such as rejecting, reducing, reusing, recycling) also do so as part of the expression of their identities or desired identities.

It has been argued that ***playfulness***, a key dimension of wellbeing, needs to be taken more seriously as a part of our future approach to sustainable consumption (Wilk, 2022). Playfulness is understood here as an engagement with fashion that is not solely characterised by exploitation and utility, i.e. using fashion and its products in established ways to achieve specific goals and values, but rather also by exploration and fun, i.e. engaging with fashion in novel ways that have uncertain and maybe non-utilitarian outcomes, but are nevertheless emotionally and socially satisfying engagements. The argument is that, as marketing and product development have made playfulness a key part of the problem of overconsumption, a novel framework for sustainability could leverage playfulness to become a part of the solution to help dissipate the common sense that sustainability is boring and worthy (ibid.). The specifics of what this might involve need more research, particularly in relation to distinct socio-economic contexts. For example, Soper’s concept of ‘Alternative Hedonism’ (2020; 2008) has been critiqued for its notably middle-class non-GDP oriented pleasures such as crafting, gardening, and being in nature (Wilk, 2022). Nonetheless, people are adaptable and imaginative in how they find or make fun in all sorts of contexts, and there is potential to mobilise this capacity to encourage sustainable consumption in future (ibid). Wilde and Underwood (2018), for instance, explore designing garments under conditions of uncertainty regarding outcomes or even the materials themselves, so as to allow for increased playfulness, curiosity, and enjoyment of the nature and experience of materials and increased co-creation. The potential for play in fashion has notably expanded in the digital space (Park and Chun, 2023). Here, fashion takes on new possibilities for creativity and self-expression beyond wearing ‘real’ clothes. These include ‘pretending to have multi-personas, fashion-themed interaction and memes in social media, voluntary play in a brand platform, sensory expansion, and departure from space–time through brand experience’ (ibid., p. 16). Such avenues for digital play offer one direction for applying the argument for mobilising playfulness to engage people in sustainable consumption.

While doing well requires a sense of hope for a sustainable future, concerns about the threat posed by climate change evoke feelings of uncertainty, anxiety, and hopelessness for many people (Landry et al., 2018; Clayton, 2020). Taking positive actions for, and through, the CE requires a sense of ***optimism*** (notions of a bright/good future) and a vision of a ***future (prospective) self*** (i.e. the capacity to imagine future outcomes and engage in preemptive change). Expressing concern and feeling hopeful about the future may motivate pro-environmental behaviour and engender trust that one’s actions can ameliorate pressing ecological dilemmas (Ojala, 2012, 2015). Conversely, hope may be underpinned by ‘wishful thinking’ and denialism, leading to complacency and inaction (Ojala, 2012). Furthermore, negative emotionality (e.g. fear) may be important in being able to identify climate change as a threat and encourage conservation behaviour (Kleres and Wettergren, 2017). Indeed, being able to acknowledge environmental dissolution and wanting to avoid negative future outcomes might motivate compensatory pro-environmental behaviour (i.e. the constructive pessimism hypothesis; Kaida and Kaida, 2016). Nonetheless, like the emotion of hope, heightened optimism (i.e. expressing positive future expectancies) may inspire and motivate people to take charge and engage in conservation behaviour (Kaida and Kaida, 2019; McAfee et al., 2019). Accordingly, ambiguity remains surrounding the relative roles of optimism and pessimism in predicting pro-environmental behaviour. While sadness has a role in motivating people in relation to environmental issues (Forgas, 2017), pessimism and fear can serve to paralyse people into inaction—the opposite of what is needed. Ultimately, therefore, sustainability must balance both optimism and pessimism.

Alongside a sense of (balanced) optimism, it is important for people to be able to picture or imagine their future self as either increasing sustainable behaviours (e.g. using repairing their garments more often) or reducing unsustainable behaviours (such as consuming less clothes). The ability to mentally simulate the future has been referred to as prospective thinking (Schacter et al., 2012), while the ability to also simulate one’s actions in the future relates to the idea of the ‘prospective-self’ and related future-oriented concepts such as ‘self-efficacy’ (Bandura, 1982): that is, the belief that one has the capabilities needed to achieve a goal or cope with a difficulty. This kind of prospective thinking involves both the retrieval of information that is stored in episodic memory (e.g. details about previous goals and habits and achievements) and more abstract, schematic and conceptual knowledge (such as envisioning general pro-environmental goals or the development of novel sustainable solutions) (D’Argembeau et al., 2011; D’Argembeau and Demblon 2012). There has been recent interest in understanding the cognitive and brain mechanisms that influence how individuals picture themselves in terms of either increasing future sustainable behaviours or decreasing unsustainable behaviours (Brevers et al., 2021), with initial findings revealing that increasing sustainable behaviours is considered as more feasible by individuals than reducing unsustainable ones. Indeed, other studies have shown that developing sustainable, ‘good’ habits might be more efficient (Galla and Duckworth, 2015; Wood, 2019) and less effortful (Inzlicht et al., 2014) than reducing unsustainable, ‘bad’ ones. Moreover, other studies have shown that leveraging longer prospective perspectives can lead to increased pro-environmental behaviours (Hershfield et al., 2014). Thus, taking into account ways in which to facilitate individuals to imagine or picture their future self and increase their self-efficacy is important in the context of the CE where sustainable behaviours represent trade-offs between current, known sacrifices and uncertain future benefits. Such conditions of future uncertainty are known to be subject to psychological factors such as high temporal discounting (e.g. devaluing future benefits as a fraction of their delay; Berry et al., 2017; Wilson and Dowlatabadi, 2007). Effectively, trying to make decisions currently while trying to think of the future of the planet encounters the same cognitive and emotional difficulties as trying to balance one’s current needs with saving for one’s pension. Therefore, technology such as augmented or virtual reality can be innovatively used in order to allow individuals to visualise and interact with alternative future consumption or environmental scenaria, including potentially avatars of their own future self.

### Being well

Being well refers to the objective and socioeconomic dimensions of wellbeing, in addition to health-promoting conditions, experiences and behaviours already discussed in relation to feeling well and doing well. A key aspect relevant to wellbeing, fashion and sustainability is ***affordability***, which in turn relates to feasibility, convenience and access. Affordability is a prerequisite for engagement with other dimensions of wellbeing in the context of sustainable fashion consumption. Sustainable fashion products and services tend to be more expensive both in monetary value and time than less sustainable options. Affordability is a consistent priority for consumers: price is regularly found to limit or override other factors involved in purchase decision making (Brandão and Costa, 2021), even among more ‘eco-conscious’ fashion consumers (Connell, 2010). That said, occasional studies of sustainable fashion consumers have also found people to be willing to pay more because they see their purchases as higher-quality and, therefore, longer-term ‘value for money’ (Lundblad and Davies, 2016). However, ‘sustainable’ practices are most likely to be enacted by everyday consumers where these are affordable, and even then, acting more sustainability (e.g. buying second hand) is not necessarily part of consumers’ decision making (McNeil and Moore, 2015).

### Consolidating the wellbeing framework for consumer experiences in the circular economy of the textile industry

**Fig. 1:**
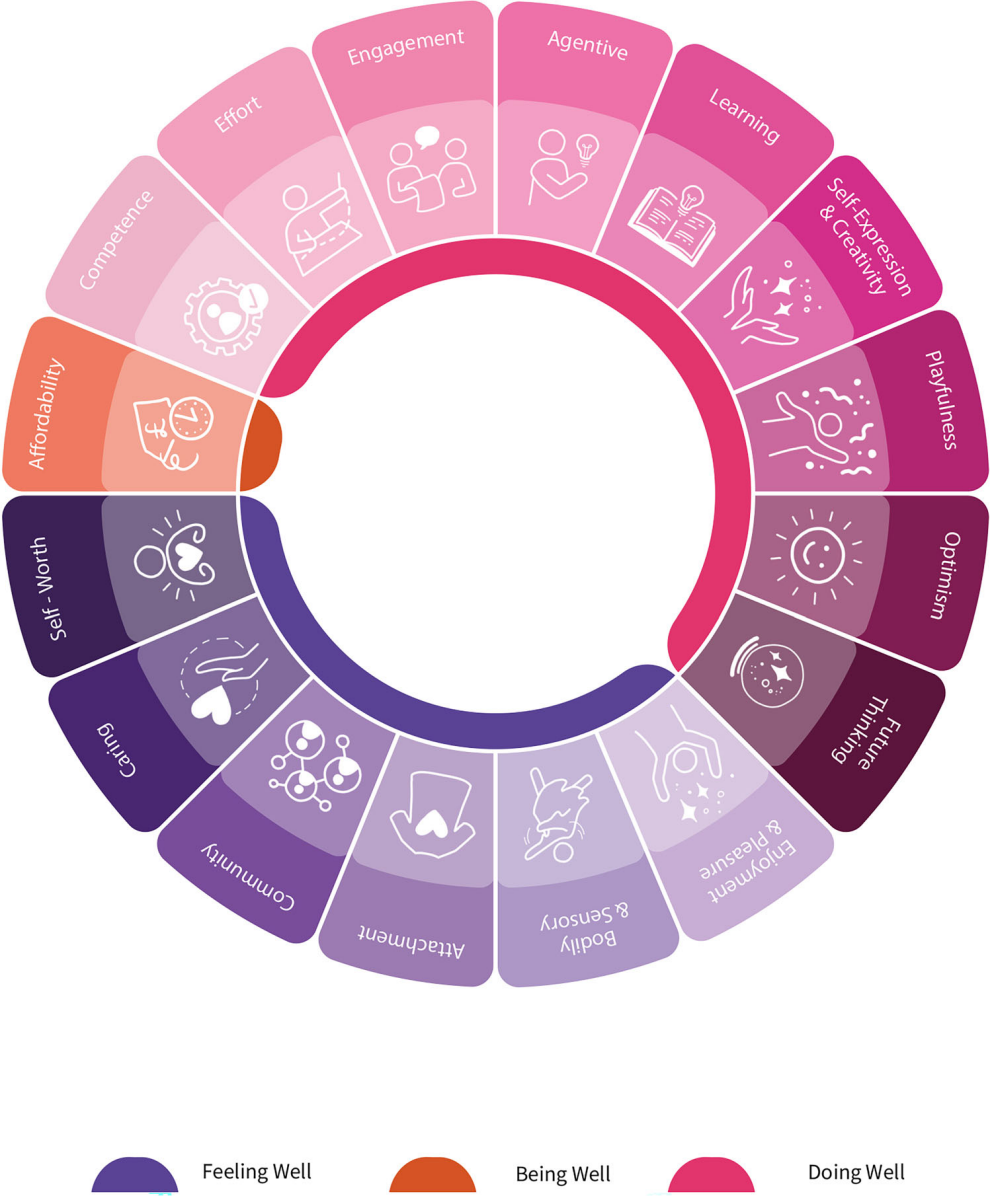
Wellbeing framework for consumer experiences in the circular economy of the textile industry. [Concept: The authors; Illustrations: Amber Anderson; Graphic design: Mary Stansfield].

In this paper, we developed a comprehensive and integrated framework for understanding wellbeing. Our key contribution to the existing literature includes identifying 16 concepts across three broad categories—being well, feeling well and doing well—that support the design of consumer experiences, products, and services in conducive ways for a circular economy. To consolidate our contribution, we created the diagram below (Fig. 1) to illustrate the framework and its concepts and maximise the impact of our findings and engage readers. This framework examines how a holistic approach to consumer wellbeing can provide social benefits through circularity, foster new consumption cultures that promote sustainable behaviour, and serve as a consumer-driven tool to advance towards zero waste through responsible and personalised participation. This framework has the potential to support practical changes in consumer-driven societies, especially in the fashion and textiles industry.

## Conclusion

This paper focuses on consumer experiences, offering a definition of wellbeing situated in the context of circular textile consumption. Through an extensive literature review, we identified sixteen concepts that can support the design of future consumer experiences, products, and services.

The theoretical Wellbeing framework for consumer experiences in the circular economy of the textile industry presented in this paper was designed according to the Textiles Circularity Centre’s working principles, which aim to redefine material value beyond cost to include socioeconomic factors. This involves significantly reducing consumption, extending the use of garments and increasing their lifespan, and minimising consumer waste through reuse, recycling, or other circular strategies. It also involves using traceable and durable materials, streamlining production processes via localisation, reducing or eliminating the use of virgin materials, and reducing land use. Consumers are central to these circular efforts.

Our literature review highlighted that while circularity extends beyond individual consumer efforts, their awareness, attitudes, and behaviours are crucial for the future circular economies of textiles, garments, and fashion. The concepts brought together in our framework are particularly useful for CE efforts, where the transition of consumers from consumption to active stakeholder roles is critical for textiles' circularity. This transition requires consumers to align their intentions with actions, beginning with an agentive attitude, active engagement in circular practices, and education about materials lifecycles, circular business models, and the various stakeholders involved.

Our research testifies that wellbeing concepts are familiar to scholars working across sustainability, CE, and fashion disciplines. Our concerted effort to integrate these concepts into a cohesive framework aims to inform this new design space of fashion product-service-experience offerings conducive to sustainable consumption. This effort has enabled us to identify ways to put these concepts into relationships in productive ways for the CE.

Changing consumer behaviour is inherently difficult, and barriers remain, however, the Wellbeing framework for consumer experiences in the circular economy of the textile industry presents a more systemic and applicable approach to fostering pro-environmental change, moving away from traditional normative frameworks. The framework is primarily conceptual, with limited empirical validation in this study, and further empirical evidence is needed to support its claims, however, the paper’s key strengths lie in its ability to create a holistic, well-synthesised framework for understanding wellbeing and its potential to drive tangible, practical changes in consumer-based societies in the context of fashion and textiles. Further empirical evidence will be provided in future research by this consortium, and we hope the academic community will further contribute to this effort.

We understand that wellbeing, while important, is but one of several dimensions forming new circular consumer experiences. Experiences that must also encompass socio, environmental and technical dimensions, as well as the product type, brand identity and business model. We attend to these dimensions elsewhere through empirical studies to further refine our framework (Jewitt et al., in review), nonetheless, we propose that wellbeing be at the heart of new circular consumer experience design. This is fundamental to design out overconsumption and promote people to benefit from the wellbeing concepts that are mapped in this paper. This will support people to derive a more plural, multifaceted wellbeing from fashion, rather than the one-dimensional satisfaction experienced through current linear consumption.

We conclude the paper by suggesting that the precise and holistic definition of wellbeing proposed in this paper can support retail and designers working in the circular textile economy to create meaningful consumer experiences centred around wellbeing. When well defined and holistic (i.e. beyond hedonism) wellbeing appears to have the potential to be coupled with material resource flow to shape sustainable consumer behaviour.

### Limitations and future research

The Textiles Circularity Centre tested some of the wellbeing dimensions conceptualised here through novel empirical design-led research in speculative ‘retail’ contexts to demonstrate how this definitional work on wellbeing can provide the basis for a design strategy to bring wellbeing into the design of meaningful circular consumer experiences. That is, how designers and brands can design wellbeing dimensions to strategically facilitate ‘circular’ sustainably-oriented behaviour. These will appear in a portfolio of publications reporting on empirical studies to follow this paper.

The Wellbeing Framework for Circular Consumer Experiences will underpin:

- design of products, services and experiences that empower consumers to become active nodes in the circular value chain, enabling responsible and personalised engagement;
- consumer-facing in-store experiences and services that ease the adoption of circular practices by amplifying couplings between the resource flow, wellbeing and satisfaction;
- design of new, digitally immersive experiences and services around apparel products, productive for textiles circularity.

Further research is required to explore how these ideas would work ‘in the wild’; that is, in the context of an existing shop with ‘real’ consumers. A key part of this development is to work with existing brands and fashion collectives. These steps are underpinned by a broader point regarding the need for longer-term empirical research, both into the lasting efficacy of individual consumption-reducing strategies borne of empirical pilot studies (e.g. see Maldini et al., 2019), and into the ideas about human wellbeing that underpin them. In addition, further research is also required to address these points across different cultural contexts. These possibilities need to be understood against the social realities (and social capital needed) to engage with such sustainable activities, that is, barriers such as convenience, time, budget, effort, skills, etc. More work is needed to support sustainable consumption at a practical everyday level that speaks to these constraints (Heinze, 2021), and into how brands and governments could better support consumers to contribute to fashion and textiles circularity (Fashion Revolution Poland, 2023).

## Data Availability

No datasets were generated or analysed during the current study.

## Footnotes

1 Evans (2019) defines consumption as a process consisting of a series of “moments” that occur during the performance of other practices. These moments include acquisition (exchange and access to goods and services), appropriation, appreciation (enjoyment during use that creates meaning and derives pleasure), devaluation, divestment (loss of attachment, meaning, or value), and disposal. Evans’s six ‘moments’ of consumption provide a useful framework, highlighting that waste processes are the inevitable ‘flip’ side of consumption.
